# The Roles of Psychological Flexibility and Perceived Parental Emotional Support in Resilience and Social Anxiety Among College Students

**DOI:** 10.3390/bs16060960

**Published:** 2026-06-10

**Authors:** Haiyan Cui, Min Xie, Shuyue Zhang

**Affiliations:** Faculty of Education, Guangxi Normal University, Guilin 541004, China; chy@stu.gxnu.edu.cn (H.C.); xm1997@stu.gxnu.edu.cn (M.X.)

**Keywords:** resilience, psychological flexibility, social anxiety, perceived parental emotional support

## Abstract

Against the backdrop of the growing prevalence of social anxiety among college students, it is of great significance to explore the internal and external protective resources of college students to prevent and buffer social anxiety. Resilience, as an important protective psychological trait, is closely associated with social anxiety. However, how resilience functions through internal and external resources of psychological flexibility and perceived parental emotional support remains to be systematically explored. Based on the stress and coping theory and related research, this study constructed a moderated mediation model. It aimed to examine the relationship between resilience and social anxiety among college students, as well as the mediating role of psychological flexibility and the moderating role of perceived parental emotional support. A cross-sectional survey was conducted among 1713 college students using a questionnaire survey method. The results showed that resilience negatively and significantly predicted college students’ social anxiety, with psychological flexibility playing a mediating role. Perceived parental emotional support moderated the relationship between resilience and psychological flexibility, as well as the relationship between resilience and social anxiety. Specifically, perceived parental emotional support strengthened the positive predictive effect of resilience on psychological flexibility, while also enhancing the direct negative predictive effect of resilience on social anxiety. This study reveals the internal mechanism through which resilience is associated with college students’ social anxiety, providing empirical evidence and practical implications for mental health education and intervention.

## 1. Introduction

Personal growth and development are inextricably linked to society, with social interaction permeating the entirety of individuals’ lives. In recent years, social anxiety has garnered widespread attention. Social anxiety refers to individuals’ excessive worry or panic about their interpersonal circumstances, a fear of negative evaluation from others, accompanied by behavioral and emotional reactions such as social avoidance, tension and inhibition, and self-denial (Watson & Friend, 1969). As one of the prevalent anxiety problems, social anxiety is particularly prominent among college students. A survey conducted in China showed that the incidence of social anxiety among college students was 47.915% (Deng & Zhang, 2026). In China, when students enter university, their social circles expand, and their interaction partners differ significantly in regional background, living habits, values, and other aspects. Complex interpersonal relationships and social environments often leave college students feeling anxious and uneasy (Ogunrinde et al., 2025). According to Erikson’s theory of psychosocial development, college students face the psychosocial crisis of intimacy versus isolation (Fanchang, 2024), and establishing stable and positive interpersonal connections is an important task for them to complete socialization. If left unaddressed, this crisis could result in strained social relationships, which in turn may increase the risks of loneliness and depression. Persistent social anxiety among college students not only impairs their current interpersonal functioning (K. Zhao et al., 2025), but also affects academic performance (Mou et al., 2024) and life satisfaction (Foroughi et al., 2022). It may even increase the risk of psychological and behavioral problems such as depressive disorders (Piao et al., 2025), substance abuse (Leigh & Clark, 2018), and suicidality (Leigh et al., 2023). Social anxiety has become one of the major factors affecting college students’ mental health and thus deserves serious attention.

The occurrence of social anxiety is linked to multiple factors (Liu et al., 2025). Most existing studies have focused on risk factors and identified potential triggers of social anxiety. These include individual factors such as negative cognitive patterns and interpersonal sensitivity (Barahmand et al., 2023; X. Li et al., 2024); family factors such as adverse family environments and maladaptive parenting styles (e.g., helicopter parenting) (Jiao et al., 2025; Wei & Mei, 2026); and social environmental factors such as negative life events (Teng et al., 2025), peer victimization (Di Tata et al., 2024), and social exclusion (Zhang et al., 2025). These research findings provide empirical foundations for understanding and intervening in social anxiety. As research continues to advance, positive psychology has increasingly focused on remediating individuals’ psychological problems while helping individuals recognize, amplify, and cultivate their own strengths, thereby promoting personal development (Seligman & Csikszentmihalyi, 2000). Meanwhile, it has been gradually recognized that focusing solely on risk factors is insufficient to fully account for individual differences in social anxiety. Some individuals seem to be able to recover effectively from negative events, while others get stuck and seem unable to shake off negative emotions (Tugade & Fredrickson, 2004). In reality, even when facing similar socially stressful situations, some individuals still maintain sound social adaptation. This indicates that positive psychological resources within individuals play a vital protective role in buffering negative emotions and resisting social anxiety. As a positive psychological trait, resilience is a protective factor for individuals in coping with adversity (Rutter, 1985) and is regarded as a key internal resource for individuals to resist social anxiety. Existing studies have found that there is a negative correlation between resilience and social anxiety, highlighting the importance of resilience factors in managing social anxiety (Jefferies et al., 2021). Resilient individuals are not only able to recover effectively from stressful experiences (Tugade & Fredrickson, 2004), but can also mobilize internal and external resources and adopt positive coping strategies when facing social threats, negative evaluations, and interpersonal stress, helping them maintain emotional balance and thereby buffering the emergence and exacerbation of social anxiety (Y. Li & Zheng, 2025). Although research has confirmed the protective effect of resilience on social anxiety, the underlying mechanisms through which resilience exerts its effect still require further clarification and in-depth exploration.

Stress and coping theory focuses on how individuals perceive and cope with stressors in the environment (Biggs & Brough, 2025). When confronted with environmental stressors, individuals exhibit responses characterized by physiological arousal and negative emotions, with anxiety being particularly typical (Folkman, 2013). This theory posits that stress arises not only from external events but also from the interaction between individuals and their surrounding environment, with its primary focus on cognitive appraisal and stress coping (Biggs et al., 2017). The degree of anxiety experienced by college students in social situations depends on whether they appraise social scenarios as “threats” or “challenges” during primary appraisal, and whether they perceive themselves as possessing sufficient coping resources during secondary appraisal. Resilience serves as an internal resource for individuals to cope with adversity and stress (Ward et al., 2020), and it is related to individuals’ appraisal of social anxiety. Furthermore, psychological flexibility reflects individuals’ ability to maintain acceptance and defusion from present-moment experiences while facing negative emotions such as anxiety and fear, and to continue taking effective actions aligned with personal values (K. M. Cherry et al., 2021). Studies have indicated that it serves as an important pathway through which resilience functions (Elliott et al., 2019; Pakenham et al., 2024). However, existing studies have not yet elucidated the specific role that psychological flexibility plays between resilience and social anxiety in college students. Meanwhile, stress and coping theory emphasizes the synergistic interaction between internal coping resources and external environmental resources (Biggs et al., 2017). Recent studies have shown that perceived parental emotional support is associated with positive emotional and mental health outcomes (Weng et al., 2025), providing individuals with emotional security and a sense of coping efficacy when facing stress, and serving as an external environmental resource for regulating social anxiety. It follows that resilience may alleviate social anxiety by activating individuals’ inherent psychological flexibility, and may also be subject to the buffering effect of individuals’ external support system (perceived parental emotional support). However, the specific roles that psychological flexibility and perceived parental emotional support play between resilience and social anxiety still remain to be further explored. Specifically, the following question needs to be addressed: How does resilience alleviate social anxiety through the internal and external resources of psychological flexibility and perceived parental emotional support?

Therefore, the present study aims to investigate the relationship between resilience and social anxiety among college students, with a focus on elucidating the roles played by psychological flexibility and perceived parental emotional support in this relationship. It seeks to provide empirical evidence and theoretical support for the active prevention and intervention of social anxiety among college students.

### 1.1. Resilience and Social Anxiety

The cognitive-behavioral model of social anxiety (Rapee & Heimberg, 1997) points out that individuals experience anxiety in social situations primarily due to negative cognition of social information and self-related cognitive biases. Social anxiety is mainly characterized by distorted self-awareness, fear of negative reactions and scrutiny from others, avoidance of social situations, and symptoms such as blushing, sweating, and increased heart rate (Morrison & Heimberg, 2013). When confronted with challenging social situations, individuals with social anxiety tend to overestimate the negative consequences of social encounters, perceive themselves as having limited control over their emotional reactions, and believe their social skills are insufficient to effectively cope with social situations (Hofmann, 2007). Among college students, social anxiety, as a typical negative emotion and psychological adaptation issue, is regarded as a psychological challenge faced in social situations. According to resilience theory, individuals with high resilience possess internal psychological resources to cope with adversities, which can buffer negative mental health outcomes such as anxiety and stress triggered by stressful events (Davydov et al., 2010; J.-L. Wang et al., 2015). Resilience refers to individuals’ ability to effectively adapt to adverse environments and rapidly restore psychological equilibrium when facing negative life events such as adversity, trauma, or stress (Southwick et al., 2014). As a protective factor of individuals, resilience enables people to mobilize internal and external resources, cope with stress effectively, and reduce the occurrence of negative emotions and behavioral problems.

Multiple studies have consistently demonstrated a significant negative correlation between resilience and social anxiety: high resilience helps alleviate social anxiety. This finding has been validated across populations including individuals with aphasia, general adults, and college students (Jefferies et al., 2021; R. Zhao et al., 2025; Atik et al., 2026). Further mechanistic research has revealed that resilience exerts an indirect effect on social anxiety through various pathways. Resilience can reduce social anxiety by enhancing emotion regulation abilities and promoting approach-oriented coping strategies, and can also alleviate social anxiety symptoms by improving perceptions of interpersonal relationships (Y. Li & Zheng, 2025; R. Zhao et al., 2025). In conclusion, resilience provides important psychological protective resources for college students, helping them effectively cope with social anxiety. Accordingly, this study proposes Hypothesis 1: Resilience negatively predicts social anxiety.

### 1.2. Psychological Flexibility as a Mediator

Psychological flexibility, as a core concept of acceptance and commitment therapy (Hayes et al., 2012b), provides a new perspective for understanding the underlying internal mechanisms of resilience. Psychological flexibility refers to individuals’ ability to adapt to the demands of changing situations, allocate and adjust psychological resources, shift cognitive perspectives, and balance conflicts among desires, needs, and different life domains (K. M. Cherry et al., 2021). It mainly consists of three key components. First, it involves confronting interference or distress (e.g., social anxiety, facing setbacks). Second, it requires taking action to manage interference or distress (e.g., persisting, accepting, tolerating). Third, it means acting in ways that align with situational demands and facilitate the pursuit of personal goals or values (Kashdan & Rottenberg, 2010; K. M. Cherry et al., 2021).

Psychological flexibility plays a crucial role in promoting college students’ mental health, including alleviating symptoms of anxiety, stress, and depression (Yao et al., 2024; Yang et al., 2025). Research on a clinical sample of adolescents has confirmed that psychological inflexibility and acceptance and committed action (as processes of psychological flexibility) are closely associated with the development of social anxiety (Figueiredo et al., 2024). Furthermore, psychological flexibility has an inherent association with the relationship between resilience and psychological adaptation outcomes. Relevant studies have indicated that psychological flexibility mediates the relationship between resilience and academic burnout among doctor of physical therapy students (J. Cherry et al., 2024), the relationship between resilience and distress and quality of life in people with multiple sclerosis (Pakenham et al., 2024), and the relationship between resilience and adjustment in Iraq/Afghanistan war veterans (Elliott et al., 2019). These studies have conceptualized psychological flexibility as a factor through which resilience exerts its effects, providing cross-population empirical support for the mediating role of psychological flexibility between resilience and psychological adaptation outcomes. However, the mediating effect of psychological flexibility between resilience and social anxiety among college students remains unclear and requires further investigation. Thus, this study proposes Hypothesis 2: Psychological flexibility mediates the relationship between resilience and social anxiety.

### 1.3. Perceived Parental Emotional Support as a Moderator

In addition to being conditioned by individual internal factors, social anxiety is also significantly affected by the family environment, especially parental emotional support. Perceived parental emotional support refers to the subjective perceptions of emotional care, understanding, acceptance, and encouragement that children experience from their parents (Weng et al., 2025). It is reflected not only in children’s perceptions of their parents’ daily care, concern, and communication, but also in their perceptions of parental support in the form of empathy, comfort, positive attention, and assistance when they are faced with stress or setbacks. Perceived parental emotional support is a protective factor for individuals’ mental health (Romm et al., 2021). Perceived parental emotional support not only reinforces individuals’ self-esteem and alleviates psychological distress (Boudreault-Bouchard et al., 2013), but also enhances their confidence in their own abilities, thereby indirectly reducing the risk of anxiety and depression (Qian et al., 2024). Meanwhile, it contributes to shaping more accurate self-perceptions and avoiding self-evaluation biases (Côté et al., 2014), further influencing individuals’ growth mindset and resilience (T. Li et al., 2026).

Moreover, perceived parental emotional support is closely related to individuals’ social participation, exerting a positive impact on their engagement in social interactions and activities (Ratcliff et al., 2024). Perceived parental emotional support promotes the functioning of resilience. On the one hand, perceived parental emotional support may enhance the positive effect of resilience on psychological flexibility. Supportive interpersonal relationships can strengthen individuals’ coping resources and improve their psychological adjustment under stressful conditions (Holahan et al., 1997), which facilitates the positive association between resilience and psychological flexibility. On the other hand, perceived parental emotional support may also buffer the relationship between resilience and social anxiety. College students reporting higher perceived parental emotional support tend to develop greater resilience when confronting stress and adverse circumstances (Cheraghian et al., 2023). When college students receive emotional validation and encouragement from their parents, their inherent resilience is more likely to exert its protective effect in social situations. In contrast, when perceived parental emotional support is insufficient, even if college students possess a certain level of resilience, the protective effect against social anxiety may be weakened. In summary, this study proposes Hypothesis 3: Perceived parental emotional support moderates the relationship between resilience and psychological flexibility, as well as the relationship between resilience and social anxiety.

### 1.4. The Current Study

**Figure 1:**
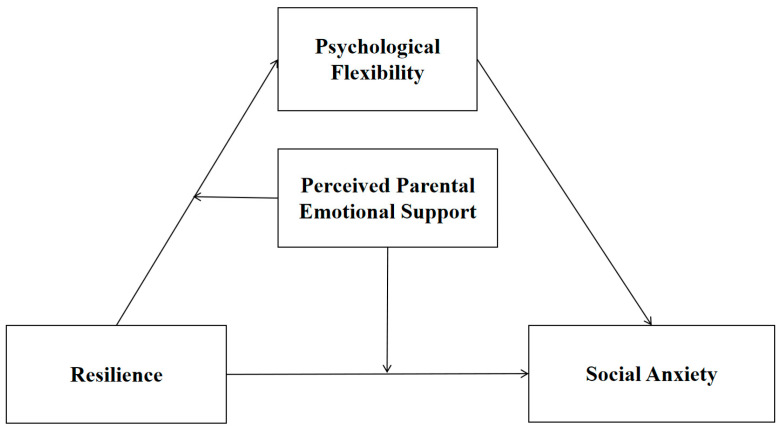
Research model.

Against the backdrop of the increasingly prominent issue of social anxiety among college students, it is of great theoretical and practical importance to deeply explore the protective factors that alleviate social anxiety in college students and to systematically examine the mechanisms through which resilience, psychological flexibility, and perceived parental emotional support function in the development of social anxiety. Theoretically, social anxiety is a result of the interaction between individuals’ internal resources and external interpersonal support systems. Although existing studies have confirmed that resilience serves as a psychological resource that protects individuals against social anxiety, the specific roles that psychological flexibility and perceived parental emotional support play in this process remain to be further explored. Practically, clarifying the synergistic effect of internal psychological processes and external family resources on college students’ social anxiety helps to promote the shift of college mental health education from passive response to active prevention, and provides practical strategies for the prevention and intervention of social anxiety among college students. Therefore, based on stress and coping theory and related research, this study constructed a moderated mediation model. It aims to examine the effect of resilience on college students’ social anxiety, the mediating role of psychological flexibility, and the moderating role of perceived parental emotional support (see Figure 1). The specific hypotheses are as follows:

**H1.**  *Resilience negatively predicts social anxiety.*

**H2.**  *Psychological flexibility mediates the relationship between resilience and social anxiety.*

**H3.**  *Perceived parental emotional support moderates the relationship between resilience and psychological flexibility, as well as the relationship between resilience and social anxiety.*

## 2. Methods

### 2.1. Participants and Procedure

This study is a cross-sectional survey that employed a convenience sampling method. Participants were full-time college students recruited from three universities in Guangdong Province, China. Inclusion criteria required participants to be full-time undergraduates spanning freshmen through seniors with intact reading, writing, and communication skills, enabling them to complete questionnaires independently. All participants took part in the survey entirely on a voluntary basis and were encouraged to raise questions or seek clarification whenever needed. Participants were excluded if they had received psychotherapy or regular psychological counseling within the preceding three months or had a clinical diagnosis of social anxiety disorder or other serious mental disorders. We recruited one teacher from each university to serve as the survey administrator. These administrators explained the research purpose and provided instructions to eligible students, and then distributed online questionnaires via a Chinese online platform, inviting voluntary participation. Informed consent was obtained from all participants before data collection. The questionnaire was completed anonymously, and the ethical principle of voluntary participation was strictly followed. Participants could withdraw from the study at any time without any adverse consequences. They were assured that the data would be used solely for academic research and kept strictly confidential. This study was approved by the Ethics Committee of Guangxi Normal University, and all research procedures complied with the requirements of the Declaration of Helsinki.

A total of 1810 questionnaires were collected. According to data preprocessing criteria, invalid questionnaires with a response time of less than 100 s and those showing obvious response inertia (i.e., selecting the same option for all items) were excluded. Finally, 1713 valid questionnaires were obtained, with an effective recovery rate of 94.64%. Participants’ ages ranged from 17 to 24 years (Mage = 20.301, SDage = 1.535). Among them, 818 were male students (47.75%) and 895 were female students (52.25%). In terms of only-child status, 425 were only children (24.81%) and 1288 had siblings (75.19%). Regarding grade level, 442 were freshmen (25.80%), 436 were sophomores (25.45%), 419 were juniors (24.46%), and 416 were seniors (24.28%).

### 2.2. Measures

#### 2.2.1. Resilience Scale

The Chinese version of the Connor–Davidson Resilience Scale (CD-RISC-10) was adopted in this study (L. Wang et al., 2010). The scale comprises 10 items (e.g., “Able to adapt to change”), rated on a 5-point Likert scale (0 = “never”, 4 = “always”). Higher total scores indicate higher levels of resilience. The results of confirmatory factor analysis (CFA) indicated an acceptable model fit (*χ*^2^ = 227.253, *df* = 30, RMSEA = 0.062, CFI = 0.959, TLI = 0.938, SRMR = 0.039). The scale yielded excellent internal consistency, with a Cronbach’s *α* of 0.917.

#### 2.2.2. Psychological Flexibility Scale

This study employed the Chinese version of the Psychological Flexibility Scale (Jiang et al., 2024). The scale comprises 15 items (e.g., “I avoid the most difficult goal-related tasks”), including three subscales: avoidance, acceptance, and harnessing. It uses a 7-point Likert scale ranging from 1 (strongly disagree) to 7 (strongly agree). The scores for the avoidance subscale were reverse-coded. For ease of interpretation, this study reverse-coded the scores of the avoidance subscale before data analysis. Higher total scores on the scale indicate greater psychological flexibility. The CFA results demonstrated an acceptable model fit (*χ*^2^ = 369.824, *df* = 74, RMSEA = 0.048, CFI = 0.973, TLI = 0.962, SRMR = 0.056). The three subscales showed good reliability, with Cronbach’s *α* values of 0.885, 0.815, and 0.881, respectively, while the total scale demonstrated excellent internal consistency with a Cronbach’s *α* of 0.948.

#### 2.2.3. Social Anxiety Scale

This study adopted the Chinese version of the Short Form of the Social Anxiety Scale originally developed by Zhou et al. and revised by Sun et al. (Sun et al., 2017; Zhou et al., 2008). The scale consists of 12 items (e.g., “I feel shy around people I don’t know”) and encompasses three dimensions: Fear of Negative Evaluation, Social Avoidance and Distress-New, and Social Avoidance and Distress-General. It uses a 5-point Likert scale (1 = “completely inconsistent”, 5 = “completely consistent”), with higher total scores indicating higher levels of social anxiety. The CFA results revealed an acceptable model fit (*χ*^2^ = 427.485, *df* = 51, RMSEA = 0.066, CFI = 0.956, TLI = 0.943, SRMR = 0.036). The three dimensions exhibited excellent reliability, with Cronbach’s *α* values of 0.899, 0.901, and 0.900, respectively, and the total scale had a Cronbach’s *α* of 0.943.

#### 2.2.4. Perceived Parental Emotional Support Scale

The Perceived Parental Emotional Support Scale (Child Version, PPESS) was used in the present study (Tang et al., 2026). The scale consists of 15 items (e.g., “Parents can immediately notice my emotional changes”) and includes four dimensions: perceived emotional bonding, perceived emotional awareness, perceived emotional resonance, and perceived emotional communication. All items are rated on a 5-point scale ranging from 1 (completely inconsistent) to 5 (completely consistent). Higher total scores indicate higher levels of perceived parental emotional support. The CFA results indicated an acceptable model fit (*χ*^2^ = 495.482, *df* = 80, RMSEA = 0.055, CFI = 0.956, TLI = 0.943, SRMR = 0.028). The four dimensions had Cronbach’s *α* values of 0.865, 0.743, 0.797, and 0.922, respectively, while the full scale had an overall Cronbach’s *α* of 0.934.

#### 2.2.5. Demographic Covariates

Gender (1 = male, 0 = female), only-child status (1 = yes, 0 = no), age, and grade (1 = freshmen, 2 = sophomores, 3 = juniors, 4 = seniors) were included as covariates in this study.

### 2.3. Statistical Analysis

Data analyses were conducted using SPSS 27.0 and Mplus 8.3. Specifically, SPSS 27.0 was adopted for statistical analyses, including scale reliability testing, descriptive statistics, correlation analysis, and Harman’s single-factor test to examine common method bias. Mplus 8.3 was used to perform confirmatory factor analysis (CFA). Since this study focuses on associations among the overall constructs of psychological flexibility, social anxiety, and perceived parental emotional support rather than dimension-specific pathways, we aggregated dimension scores to avoid excessive model complexity that would outstrip the statistical power of the current sample. The PROCESS 4.0 macro was employed to test the mediating effects and moderated mediating effects. Parameter estimation was performed using the bias-corrected bootstrapping method with 5000 resamples, and a 95% confidence interval (95% CI) that did not include zero was considered statistically significant. All analyses controlled for the demographic variables of college students’ gender, only-child status, age, and grade.

## 3. Results

### 3.1. Common Method Bias

Given that all variable data were collected via college students’ self-report, Harman’s single-factor test was performed to examine common method bias. The results identified seven factors with eigenvalues greater than 1. The first factor explained 27.449% of the total variance, which was below the critical threshold of 40%. These findings indicated that severe common method bias was not present in the present study.

### 3.2. Descriptive Statistics and Correlation Matrix

The means (M), standard deviations (SD), and Pearson correlation coefficients for the main variables are presented in Table 1. The results indicated that psychological resilience was positively and significantly correlated with psychological flexibility (*r* = 0.228, *p* < 0.001) and perceived parental emotional support (*r* = 0.429, *p* < 0.001), yet negatively and significantly linked to social anxiety (*r* = −0.329, *p* < 0.001). Psychological flexibility was negatively and significantly correlated with social anxiety (*r* = −0.247, *p* < 0.001) and positively and significantly correlated with perceived parental emotional support (*r* = 0.180, *p* < 0.001). Perceived parental emotional support was negatively and significantly correlated with social anxiety (*r* = −0.316, *p* < 0.001). These correlation analysis results provided preliminary support for the subsequent tests of the mediating and moderated mediating effects.

### 3.3. Testing of the Mediating Role of Psychological Flexibility

The mediating effect of psychological flexibility was examined using Model 4 of the PROCESS 4.0 macro developed by Andrew F. Hayes. Covariates including gender, only-child status, age, and grade were controlled in the mediation analysis. The results are presented in Table 2. The direct effect of resilience on college students’ social anxiety was significant (*β* = −0.378, *p* < 0.001). Resilience significantly and positively predicted psychological flexibility (*β* = 0.396, *p* < 0.001), and psychological flexibility significantly and negatively predicted college students’ social anxiety (*β* = −0.145, *p* < 0.001). Further bootstrap analysis revealed that the mediating effect of psychological flexibility was −0.057, with the 95% CI of [−0.078, −0.039]. Since the confidence interval did not include zero, the mediating effect was statistically significant, accounting for 13.103% of the total effect (−0.435). Specific results are presented in Table 2. Therefore, psychological flexibility mediated the relationship between resilience and college students’ social anxiety. Both H1 and H2 were supported.

### 3.4. Testing of Moderated Mediation Model of Perceived Parental Emotional Support

To further examine the moderating role of perceived parental emotional support in the mediation model, Model 8 of the PROCESS 4.0 macro was employed to test the moderated mediating effect. The results are presented in Table 3. First, both resilience (*β* = 0.311, *p* < 0.001) and perceived parental emotional support (*β* = 0.156, *p* < 0.001) significantly and positively predicted psychological flexibility. The interaction term between resilience and perceived parental emotional support also significantly predicted psychological flexibility (*β* = 0.159, *p* < 0.001), with the 95% CI of [0.069, 0.250] that did not include zero. This indicates that perceived parental emotional support significantly moderated the relationship between resilience and psychological flexibility. The index of moderated mediation was −0.020, with a 95% bootstrap confidence interval of [−0.040, −0.004], which does not contain zero, indicating a significant moderated mediation effect. Second, resilience (*β* = −0.247, *p* < 0.001), psychological flexibility (*β* = −0.124, *p* < 0.001), and perceived parental emotional support (*β* = −0.276, *p* < 0.001) all significantly and negatively predicted social anxiety. The interaction term between resilience and perceived parental emotional support also significantly predicted social anxiety (*β* = −0.193, *p* < 0.01), with the 95% CI of [−0.263, −0.124] that did not include zero. This suggests that perceived parental emotional support significantly moderated the relationship between resilience and social anxiety.

**Figure 2:**
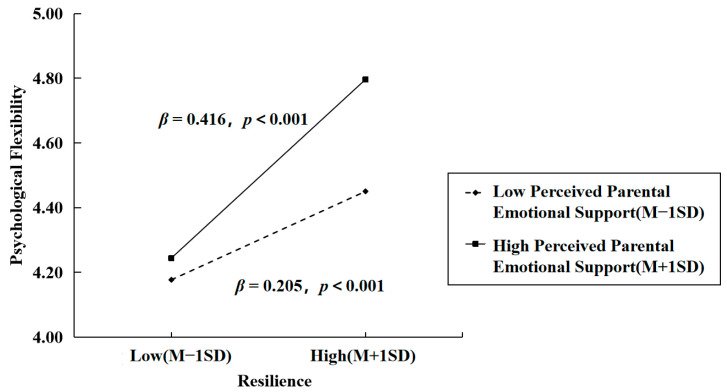
Relationship between resilience and psychological flexibility at different levels of perceived parental emotional support.

**Figure 3:**
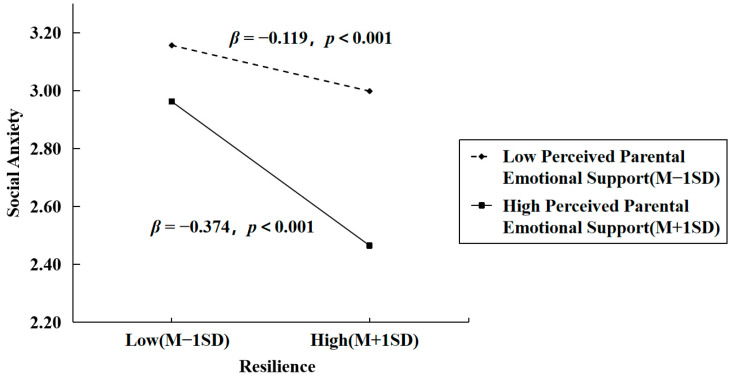
Relationship between resilience and social anxiety at different levels of perceived parental emotional support.

To clarify the pattern of the moderating effect, a simple slope analysis was conducted. Perceived parental emotional support was divided into high (*M* + 1 *SD*) and low (*M* − 1 *SD*) groups to examine the predictive effects of resilience on psychological flexibility and social anxiety at different levels of perceived parental emotional support. First, the positive predictive effect of resilience on psychological flexibility was significantly stronger in the high perceived parental emotional support group (*simple slope* = 0.416, *p* < 0.001) than in the low perceived parental emotional support group (*simple slope* = 0.205, *p* < 0.001). This indicates that perceived parental emotional support enhanced the facilitative effect of resilience on psychological flexibility, as presented in Figure 2. Second, the negative predictive effect of resilience on social anxiety was significantly stronger in the high perceived parental emotional support group (*simple slope* = −0.374, *p* < 0.001) than in the low perceived parental emotional support group (*simple slope* = −0.119, *p* < 0.001). This suggests that perceived parental emotional support strengthened the direct protective effect of resilience on social anxiety, as presented in Figure 3.

## 4. Discussion

This study constructed and tested a moderated mediation model with college students as participants, systematically examining the mediating role of psychological flexibility in the relationship between resilience and social anxiety, as well as the moderating role of perceived parental emotional support in the relationships between resilience and psychological flexibility, and between resilience and social anxiety. The findings reveal the internal mechanisms and boundary conditions underlying the impact of resilience on college students’ social anxiety. Additionally, the results offer practical implications for mental health education and intervention among college students, providing guidance for the development of mental health support strategies to alleviate social anxiety and improve college students’ mental health.

### 4.1. The Relationship Between Resilience and Social Anxiety

This study found that resilience significantly and negatively predicts social anxiety among college students, indicating that college students with higher levels of resilience tend to have lower levels of social anxiety. This finding is consistent with previous research (Jefferies et al., 2021; Y. Li & Zheng, 2025; R. Zhao et al., 2025). College students are frequently exposed to complex social scenarios, such as classroom interactions, club activities, interactions with the opposite sex, and workplace internships. In addition, they face escalating responsibilities, including academic performance, family obligations, time and financial management, future planning, and the pressure to meet both personal and societal expectations, all of which make them highly susceptible to social anxiety (Rastogi et al., 2025; Tan et al., 2023). Resilience, as a positive psychological resource, is often accompanied by positive psychological traits such as self-efficacy and optimism (Garcia-Dia et al., 2013; Schäfer et al., 2023). These positive qualities enable individuals to effectively bounce back from stressful experiences. Among these traits, the optimism inherent in resilience leads college students to hold more positive expectations about social outcomes and reduce excessive focus on potential negative evaluations. Meanwhile, college students with high resilience, by establishing and maintaining healthy self-esteem and strong self-efficacy (Rutter, 1987), tend to believe in their ability to cope with uncertainties and potential negative evaluations in social interactions. When confronted with stressful social events such as social embarrassment, interpersonal rejection, or communication setbacks, college students with high resilience are able to quickly restore psychological equilibrium, activate effective emotion regulation strategies (Tugade & Fredrickson, 2004), and proactively adopt adaptive coping methods, such as active communication and self-acceptance (Leipold & Greve, 2009). These behaviors effectively buffer the negative impact of social stress. Collectively, these characteristics reduce college students’ threat appraisal of social situations, thereby alleviating their anxiety experiences. In contrast, college students with low resilience are more prone to feelings of helplessness and frustration when facing social pressure. They often hold negative expectations about social outcomes, amplify their own sense of social discomfort, and ultimately exhibit higher levels of social anxiety.

### 4.2. Mediating Effect of Psychological Flexibility

This study further reveals that psychological flexibility plays a mediating role in the relationship between resilience and social anxiety among college students. According to stress and coping theory (Biggs et al., 2017), social anxiety is a negative emotional outcome that arises when individuals, during the primary appraisal stage of social situations, evaluate social interactions as harmful threats, underestimate their own social coping abilities, and adopt rigid coping strategies such as avoidance and suppression (Hofmann, 2007). During the secondary appraisal stage, individuals assess the resources they can mobilize and their coping abilities to adapt to the stressful situation. College students with high resilience are better able to flexibly select regulatory strategies when facing stress, adjusting their coping methods according to the demands of the social context. As the capacity to allocate psychological resources and adapt to environmental shifts (Gloster et al., 2011), psychological flexibility plays a mediating role. It operates by refining individuals’ threat judgments of socially stressful situations, integrating psychological resources, reducing extreme and catastrophizing negative cognitive biases, and reframing social challenges as opportunities for personal growth (Hayes et al., 2012a). Even when college students experience negative emotions such as nervousness or self-doubt in social contexts, they can activate psychological flexibility driven by resilience. This enables them to adopt an open stance toward negative emotions, stay focused on present social goals, break free from the constraints of psychological inflexibility (Figueiredo et al., 2024), and flexibly employ adaptive coping strategies aligned with situational demands (Yang et al., 2025). These strategies include directly solving problems, seeking social support, positively reappraising the situation, accepting current circumstances, and adaptively expressing stress-related emotions (Carver et al., 1989; Marroquín et al., 2017). When a particular coping strategy proves ineffective, a flexible psychological state allows for timely strategy switching rather than fixation on a single approach, thereby accumulating successful coping experiences in social interactions. Therefore, the protective effect of resilience on social anxiety is largely achieved by enhancing psychological flexibility. Specifically, college students with high resilience, when facing uncertainty and potential negative evaluation in social situations, are more inclined to adopt an accepting rather than avoidant coping attitude. They can perceive and accommodate their inner anxiety experiences in a more flexible manner and adjust their social behavior flexibly under value guidance, thereby reducing their levels of social anxiety.

### 4.3. Moderating Effect of Perceived Parental Emotional Support

This study found that perceived parental emotional support moderates the relationships between resilience and psychological flexibility, as well as between resilience and social anxiety. Specifically, perceived parental emotional support enhances the positive predictive effect of resilience on psychological flexibility and strengthens the direct negative predictive effect of resilience on social anxiety. This finding deepens the understanding of the boundary conditions underlying the functional role of resilience. According to the conservation of resources theory (Hobfoll, 1989), when individuals’ internal resources (e.g., resilience) are inadequate, external resources (e.g., perceived parental emotional support) can serve as a compensatory mechanism. Perceived parental emotional support provides college students with a secure psychological foundation (Waters & Cummings, 2000), and alleviates the risk of psychological rigidity stemming from low resilience. Accordingly, perceived parental emotional support exerts a critical moderating role in the positive association between resilience and psychological flexibility. When college students perceive adequate parental emotional support, they tend to develop a more positive self-perception (Z. Wang et al., 2019). With adequate perceived parental emotional support, students who experience setbacks in social situations can quickly restore psychological capital through emotional replenishment from the family. This enables them to actively adopt flexible coping strategies, bravely experiment with new interpersonal behavioral patterns, and accept uncomfortable internal experiences without resorting to avoidance. Therefore, the boosting effect of resilience on psychological flexibility becomes more pronounced under high levels of perceived parental emotional support.

Furthermore, high perceived parental emotional support amplifies the protective effect of resilience against social anxiety. This result aligns with the core tenets of the buffering theory of social support (Alloway & Bebbington, 1987), which emphasizes that supportive interpersonal relationships relieve individual stress and promote psychological and physical health by offering emotional comfort, informational guidance, and practical assistance. Notably, the function of perceived parental emotional support and the prevalence of social anxiety are inevitably shaped by distinct cultural contexts; collectivistic cultural values prevalent in Chinese society attach great importance to family bonding and parental care, which makes parental emotional support exert more far-reaching impacts on college students’ mental development compared with individualistic cultural backgrounds. When college students experience social frustrations or anxiety, parents who offer high emotional support can provide timely emotional comfort and encouragement, helping them reintegrate and replenish psychological resources. Perceived parental emotional support effectively buffers social stress (Perry et al., 2021). Such support reinforces the protective effect of resilience, allowing internal strengths to be fully utilized. Conversely, under low perceived parental emotional support or when students face emotional neglect, their resilience is more likely to be depleted by persistent loneliness, leaving them in a situation of being strong yet isolated. The lack of a secure emotional foundation also weakens the buffering effect of resilience against social anxiety.

### 4.4. Implications and Limitations

The findings of this study contribute to deepening the understanding of the influencing mechanisms of social anxiety among college students and have practical implications for preventing and alleviating social anxiety in college students, as well as promoting mental health education. First, resilience is a key target for social anxiety intervention (Connor & Davidson, 2003), and enhancing college students’ resilience should become a central goal of mental health education. Resilience training programs (Leppin et al., 2014), such as stress inoculation training, cognitive reappraisal training, and strengths identification and utilization, can help strengthen students’ psychological resources for coping with social stress. Second, cultivating psychological flexibility can serve as an effective entry point for intervening in social anxiety. Group training based on acceptance and commitment therapy (Gloster et al., 2020; Hayes et al., 2012a) can help students learn to accept anxious experiences, clarify personal values, and commit to value-aligned actions, thereby breaking the vicious cycle of anxiety and avoidance. Third, the importance of parental emotional support should not be overlooked. Mental health practitioners in universities can actively communicate with parents to help them understand the role of emotional support in college students’ mental health and master effective skills for emotional expression and support. For students receiving insufficient parental emotional support, alternative resources including peer support (Scanlon et al., 2020) and teacher support (Ruzek et al., 2016) may make up for inadequate family emotional provision.

This study has several limitations that should be considered when interpreting the findings. First, given the cross-sectional design of the present study, causal inferences drawn from the observed associations should be interpreted cautiously, since cross-sectional data fail to confirm definitive causal links between variables. Future research could adopt longitudinal designs or multi-time-point tracking methods to further examine the causal effects of relevant predictors on social anxiety. Second, all variables were measured via college students’ self-reports, which may introduce common method bias and social desirability bias. Although results from the Harman single-factor test were acceptable, and a series of procedural remedies including anonymous survey completion and standardized pre-survey instructions were implemented during data collection to reduce such biases, residual biases may still persist. Future studies may adopt multi-source assessment approaches combining peer ratings, parental evaluations, and behavioral experimental indicators with self-reported questionnaires to effectively minimize single-method bias and improve data objectivity and methodological rigor. Third, all participants were recruited solely from universities across Guangdong Province, China, limiting sample representativeness and requiring cautious extrapolation of the results. Given potential regional disparities in cultural contexts, educational settings, and parental rearing practices, the present results should be generalized to college students from other domestic or overseas regions with great caution. Future studies should validate these conclusions using samples from a wider range of universities and diverse geographic regions to strengthen the external validity and generalizability of the findings. Fourth, the present study only examined perceived parental emotional support as a specific type of family resource. Future research could extend its scope to other interpersonal resources, such as peer support and teacher support, to comprehensively depict the interaction between external resources and individual internal resources.

## 5. Conclusions

The results of this study indicate that resilience can significantly and negatively predict social anxiety among college students: the higher the level of resilience among college students, the lower their level of social anxiety. This finding verifies the important protective role of resilience as an intrinsic positive psychological resource in resisting social anxiety. Meanwhile, psychological flexibility plays a mediating role in the relationship between resilience and social anxiety among college students. Resilience not only directly alleviates college students’ social anxiety but also indirectly reduces their social anxiety levels by enhancing psychological flexibility, which serves as an internal pathway linking resilience to social anxiety. In addition, perceived parental emotional support moderates both the relationship between resilience and psychological flexibility, and the relationship between resilience and social anxiety. Specifically, under conditions of high perceived parental emotional support, the promoting effect of resilience on psychological flexibility becomes more pronounced, and the direct protective effect of resilience on social anxiety is also strengthened. This finding highlights the value of perceived parental emotional support in optimizing the psychological mechanisms, providing empirical support for the prevention and intervention of social anxiety among college students.

## Figures and Tables

**Table 1:** Descriptive statistics and correlation matrix for each variable (*N* = 1713).

Variables	1	2	3	4	5	6	7	8
1 Gender	—							
2 Only-child Status	0.014	—						
3 Age	0.094 ***	−0.128 ***	—					
4 Grade	0.079 **	−0.091 ***	0.854 ***	—				
5 Resilience	−0.008	−0.074 **	0.035	0.035	—			
6 Psychological Flexibility	−0.277 ***	0.055 *	−0.017	−0.037	0.228 ***	—		
7 Social Anxiety	0.053 *	0.063 **	−0.034	−0.013	−0.329 ***	−0.247 ***	—	
8 Perceived Parental Emotional Support	−0.040	−0.025	0.011	0.006	0.429 ***	0.180 ***	−0.316 ***	—
M	-	-	20.301	2.472	35.614	4.447	2.859	3.672
SD	-	-	1.535	1.119	0.664	1.131	0.888	0.659

* *p* < 0.05, ** *p* < 0.01, *** *p* < 0.001. Gender (1 = male, 0 = female) and only-child status (1 = yes, 0 = no) are dummy variables.

**Table 2:** The mediating effect of psychological flexibility between resilience and social anxiety.

Variables	Model 1 (Psychological Flexibility)		Model 2 (Social Anxiety)		Model 3 (Social Anxiety)	
	***β***	***t***	***β***	***t***	***β***	***t***
Resilience	0.396	10.284 ***	−0.378	−12.193 ***	−0.435	−14.233 ***
Psychological Flexibility			−0.145	−7.655 ***		
Gender	−0.627	−12.251 ***	0.002	0.038	0.092	2.271 *
Only-child Status	0.206	3.458 **	0.099	2.118 *	0.069	1.460
Age	0.067	2.101 *	−0.035	−1.405	−0.045	−1.766
Grade	−0.095	−2.170 *	0.037	1.079	0.051	1.458
*R* ^2^	0.136		0.143		0.114	
*F*	53.569 ***		47.475 ***		43.772 ***	

* *p* < 0.05, ** *p* < 0.01, *** *p* < 0.001.

**Table 3:** The moderated mediation effect of perceived parental emotional support.

Variables	Model 1 (Psychological Flexibility)		Model 2 (Social Anxiety)	
	***β***	***t***	***β***	***t***
Resilience	0.311	7.265 ***	−0.247	−7.410 ***
Psychological Flexibility			−0.124	−6.670 ***
Perceived Parental Emotional Support	0.156	3.675 ***	−0.276	−8.423 ***
Resilience × Perceived Parental Emotional Support	0.159	3.454 ***	−0.193	−5.453 ***
Gender	−0.604	−11.834	−0.017	−0.409
Only-child Status	0.212	3.584	0.088	1.932
Age	0.067	2.119	−0.036	−1.480
Grade	−0.094	−2.161	0.037	1.100
*R* ^2^	0.148		0.189	
*F*	42.290 ***		49.542 ***	

*** *p* < 0.001.

## Data Availability

The original contributions presented in this study are included in the article. Further inquiries can be directed to the corresponding author.
